# TGFβ-mediated expression of TGFβ-activating integrins in SSc monocytes: disturbed activation of latent TGFβ?

**DOI:** 10.1186/s13075-020-2130-5

**Published:** 2020-03-06

**Authors:** A. van Caam, J. Aarts, T. van Ee, E. Vitters, M. Koenders, F. van de Loo, P. van Lent, F. van den Hoogen, R. Thurlings, M. C. Vonk, P. M. van der Kraan

**Affiliations:** 1 Experimental Rheumatology, Radboudumc, Geert Grooteplein 28, 6525 GA Nijmegen, The Netherlands; 2 Department of Rheumatology, Radboudumc, Nijmegen, The Netherlands

**Keywords:** Systemic sclerosis, Transforming growth factor β, Integrin, Monocyte

## Abstract

**Introduction:**

The pathophysiology of systemic sclerosis (SSc) is closely linked to overactive TGFβ signaling. TGFβ is produced and circulates in latent form, making its activation crucial for signaling. This activation can be mediated via integrins. We investigated the balance between active and latent TGFβ in serum of SSc patients and investigated if this correlates with integrin expression on monocytes.

**Methods:**

A TGFβ/SMAD3- or BMP/SMAD1/5-luciferase reporter construct was expressed in primary human skin fibroblasts. Both acidified and non-acidified sera of ten SSc patients and ten healthy controls were tested on these cells to determine total and active TGFβ and BMP levels respectively. A pan-specific TGFβ1/2/3 neutralizing antibody was used to confirm TGFβ signaling. Monocytes of 20 SSc patients were isolated using CD14+ positive selection, and integrin gene expression was measured using qPCR. Integrin expression was modulated using rhTGFβ1 or a small molecule inhibitor of TGFBR1: SB-505124.

**Results:**

SSc sera induced 50% less SMAD3-reporter activity than control sera. Serum acidification increased reporter activity, but a difference between healthy control and SSc serum was no longer observed, indicating that total TGFβ levels were not different. Addition of a pan-specific TGFβ1/2/3 neutralizing antibody fully inhibited SMAD3-reporter activity of both acidified and not-acidified control and SSc sera. Both HC and SSc sera induced similar SMAD1/5-reporter activity, and acidification increased this, but not differently between groups. Interestingly, expression of two integrin alpha subunits *ITGA5* and *ITGAV* was significantly reduced in monocytes obtained from SSc patients. Furthermore, *ITGB3*, *ITGB5*, and *ITGB8* expression was also reduced in SSc monocytes. Stimulation of monocytes with TGFβ1 induced *ITGA5* and *ITGAV* but lowered *ITGB8* expression, whereas the use of the TGFβ receptor inhibitor SB-505124 had the opposite effect.

**Conclusion:**

Total TGFβ serum levels are not different between SSc patients and controls, but TGFβ activity is. This coincides with a reduced expression of TGFβ-activating integrins in monocytes of SSc patients. Because TGFβ regulates expression of these integrins in monocytes, a negative feedback mechanism possibly underlies these observations.

## Introduction

Systemic sclerosis (SSc) is a severe auto-immune disease, characterized by vasculopathy, inflammation, and fibrosis of connective tissues. This fibrosis affects both skin and visceral organs and can (eventually) result in loss of organ function. To date, treatment of SSc consist mostly of immunomodulation with variable results. A disease-modifying therapy has not yet been identified, partly due to a lack of understanding of SSc pathophysiology.

The excessive fibrosis in SSc is thought to arise from an imbalance in extracellular matrix (ECM) production and degradation. In many cell types, an important regulator of ECM production and degradation is transforming growth factor β (TGFβ) [1, 2]. TGFβ is a growth factor that signals via the intracellular SMAD-dependent and SMAD-independent pathways to, for example, activate production of ECM genes such as *collagen type 1* and *fibronectin*. Furthermore, TGFβ is also an important regulator of endothelial cell biology, and dysregulated TGFβ signaling is linked to formation of telangiectasia and pulmonary arterial hypertension, two forms of vasculopathy observed in SSc. Therefore, TGFβ has been suggested to play a pivotal role in SSc pathophysiology [1].

TGFβ is produced and circulates in latent form, packaged in a complex consisting of latency associated peptide (LAP) and latent TGFβ binding proteins (LTBPs). Due to this production in inactive form, an activation step is required to initiate signaling. Important activators of latent TGFβ are integrins. Integrins are transmembrane cell-matrix adhesion receptors that consist of a heterodimer of α and β subunits. Specific integrins, like αVβ5, αVβ6, and αVβ8, bind LAP/LTBP and activate TGFβ by either pulling the latency complex apart using mechanical force or by presenting this complex to (membrane-bound) metalloproteases such as MMP14 which degrade the LAP/LTBP complex. The importance of integrins for TGFβ activation is illustrated by the phenotype of *itgb6* (integrin β6) and *itgb8* (integrin β8) knockout mice, which resembles that of *TGFB1* or *TGFB3* knockout mice, e.g., these mice develop severe auto-immunity [3].

Recently, human monocytes (and macrophages) have been demonstrated to be able to lower pro-inflammatory responses via integrin-mediated activation of latent TGFβ [4]. This ability to activate latent TGFβ was even further enhanced after differentiation into alternatively activated macrophages using M-CSF. Because monocytes and alternatively-activated macrophages are linked to fibrosis and SSc [5, 6], and the likely importance of TGFβ for SSc phenotype, we investigated in this paper the latency of TGFβ in SSc blood and the expression of TGFβ-activating integrins on monocytes, to identify the role this cell type can play in excessive TGFβ signaling in SSc.

## Materials and methods

### Human material

Blood was obtained from SSc patients and healthy controls (Table 1) after informed consent according to the permission granted by the local ethics committee (study number: NL57997.091.16).

**Table 1:** Patient characteristics

	SSc patients (*N* = 10)	Healthy controls (*N* = 10)
Age (years)		
Median	60.5	60.5
Interquartile range	53.75–69 (15.25)	53.75–68.25 (14.50)
Minimum and maximum	44–70	44–70
Males	5	5
Females	5	5

### Serum isolation

Blood was drawn using 21G *×* ¾″ ×  7″ BD Safety-Lok^tm^ vacutainers (Becton, Dickinson and Company, USA) in 8.5 ml BD Vacutainer® SST™ II Advance Tubes (BD, USA). Blood was allowed to cloth for 30 min at RT, whereafter tubes were centrifuged for 10 min at RT at 1000*×g*.

To activate serum components (performed according to [7]), 30 μL serum was added to 200 μL DMEM (Gibco) with additional 0.1% w/v Bovine Serum Albumin (BSA) (Sigma-Aldrich). Subsequently, 10 μL 4 M HCl (Merck) was added, and samples were incubated on a roller for 1 h at 4 °C. Hereafter, acid was neutralized using 10 μL 4 M NaOH (Emsure®, Sigma-Aldrich). Non-acidified sera were treated by simultaneously adding 10 μL 4 M HCl and 10 μL 4 M NaOH. Acidified sera were used the very same day**.**

### Isolation of monocytes

Blood was drawn using EDTA as anticoagulant and diluted 1:1 in PBS containing 1.5% v/v Anticoagulant Citrate Dextrose Solution, Solution A (ACD-A). With the use of a Ficoll-Paque plus (GE healthcare) density gradient, PBMCs were obtained. PBMCs were washed extensively with PBS containing ACD-A to remove platelets. To isolate monocytes, positive selection for CD14 was performed using MagniSort™ Human CD14 Positive Selection Kit (eBiosciences) according to the manufacturer’s protocol. This isolation yields a typical purity of 95%. After isolation, cells were seeded in polystyrene 96 wells plates at a density of 3 × 10^5^/cm^2^ in DMEM (Gibco) supplied with sodium pyruvate and penicillin/streptomycin (p/s).

### RNA isolation and qPCR

TRI-reagent (Sigma-Aldrich, Germany) was added to 5 × 10^5^ (freshly isolated) monocytes. Subsequently, chloroform was added and samples were centrifuged at 4 °C at 11,600×*g* for 15 min. The aqueous phase was transferred and incubated at RT with an equal amount of isopropanol for 10 min. Hereafter, the samples were centrifuged for 10 min at 11,600×*g* at 4 °C. The resulting pellets were rinsed twice with 75% ethanol in H_2_O and dried under vacuum for 10 min. After adding ultrapure water, the RNA concentration was measured using a NanoDrop photospectrometer (Thermo Scientific, USA). Subsequently, 1 μg of RNA was used in a single-step reverse transcriptase PCR using oligo dT primer and M-MLV Reverse Transcriptase (Life Technologies, USA). The obtained cDNA was diluted 20 times using ultrapure water, and gene expression was measured using 0.2 μM of validated cDNA-specific primers (see Table 2) (Biolegio, the Netherlands) in a quantitative real time polymerase chain reaction (qPCR) using SYBR green master mix (Applied Biosystems). Relative gene expression (−ΔCt) was calculated using four reference genes: *GAPDH*, *HPRT*, *TBP*, and *RPS27A*.

**Table 2:** Primer sequences used in this study

Gene	Forward primer 5′→3′	Reverse primer 5′→3′
GAPDH*	ATCTTCTTTTGCGTCGCCAG	TTCCCCATGGTGTCTGAGC
HPRT*	CCTGGCGTCGTGATTAGTGA	TCTCGAGCAAGACGTTCAGT
TBP*	GCTTCGGAGAGTTCTGGGATTG	GCAGCAAACCGCTTGGGATTA
RPS27A*	TGGCTGTCCTGAAATATTATAAGGT	CCCCAGCACCACATTCATCA
*ITGA5*	ACTCAACTGCACCACCAATCA	CTCCGGGCATTTCAGGATCTG
*ITGAV*	AGCGGGACCATCTCATCACT	TGAGCAACTCCACAACCCAAA
*ITGB1*	TGGTGTGGTTGCTGGAATTG	TTTTCACCCGTGTCCCATTTG
*ITGB3*	CCGGCCAGATGATTCGAAGA	TGCTCCACAGATCATCCTTCA
*ITGB5*	TGGGGAGATGTGTGAGAAGTG	GCACTCGACGCAATCTCTCT
*ITGB6*	CTGGTGTGCTCAGGAGAATTT	CTTGGGAGACAGGGTTTTCGA
*ITGB8*	TTGCTGCTGGTGATGACAGAT	GGTGTTCCATGGTTGTCGATTT

*Reference genes

### CAGA_12_-luciferase and BRE-luciferase bioassays

Primary human skin fibroblasts were obtained by placing 3.5 mm^2^ forearm skin biopsies of healthy donors in 1 ml DMEM medium containing penicillin/streptomycin, pyruvate, and 20% FCS and culturing these biopsies for 2 weeks (i.e., spontaneous outgrowth). Medium was partially refreshed every third day. After 2 weeks, attached cells were harvested using trypsin and passaged six more times until stored in liquid nitrogen. For each bioassay, a fresh vial of these cells was used, which were cultured using DMEM containing p/s, sodium pyruvate, and 10% FCS, at 37 °C and 5% CO_2_. These cells were seeded in 96-wells plates at 5 × 10^4^ cells/cm^2^ and transfected under serum-free conditions with either a pSMAD3-responsive luciferase construct (CAGA_12_-luciferase [8]) or a pSMAD1/5-responsive luciferase construct (BRE-luciferase [9]) using an adenovirus in a multiplicity of infection of 50 or 10, respectively. After 8 h of serum starvation, cells were stimulated with 100 μl 10% human serum in complete DMEM for 16 h. Subsequently, all medium was removed, and cells were lysed using 30 μl Reporter Lysis Buffer (Promega). Hereafter, 30 μl Bright-Glo™ (Promega) was added at RT, and after 1 min, luciferase activity was measured using a CLARIOstar™ luminometer (BMG Labtech). Transfection efficiency was normalized based on GFP expression, which was driven by an expression cassette present in the CAGA_12_-luciferase virus.

### Inhibition of TGFβ

To inhibit TGFβ receptor signaling, the ALK4/5/7-kinase inhibitor SB-505124 (Sigma) was used in a concentration of 5 μM. DMSO was used as solvent control. Cells were pre-incubated with the inhibitor for 1 h before use. To neutralize TGFβ1, TGFβ2, and TGFβ3, a monoclonal mouse IgG1 antibody (MAB1835, R&D systems) was used in a concentration of 5 μg/ml. Mouse IgG1 was used as the isotype control.

### Statistics

All quantitative data are expressed as a mean of multiple repeats ± SD. For every analysis, data was checked for normality using the Shapiro-Wilk test. Student’s *t* test or one-way analysis of variance (ANOVA) with Tukey’s multiple comparison post-test was used to determine significance. The statistical analyses were performed using GraphPad Prism 5.0 software.

## Results

### Lowered TGFβ bioactivity in SSc serum

**Fig. 1:**
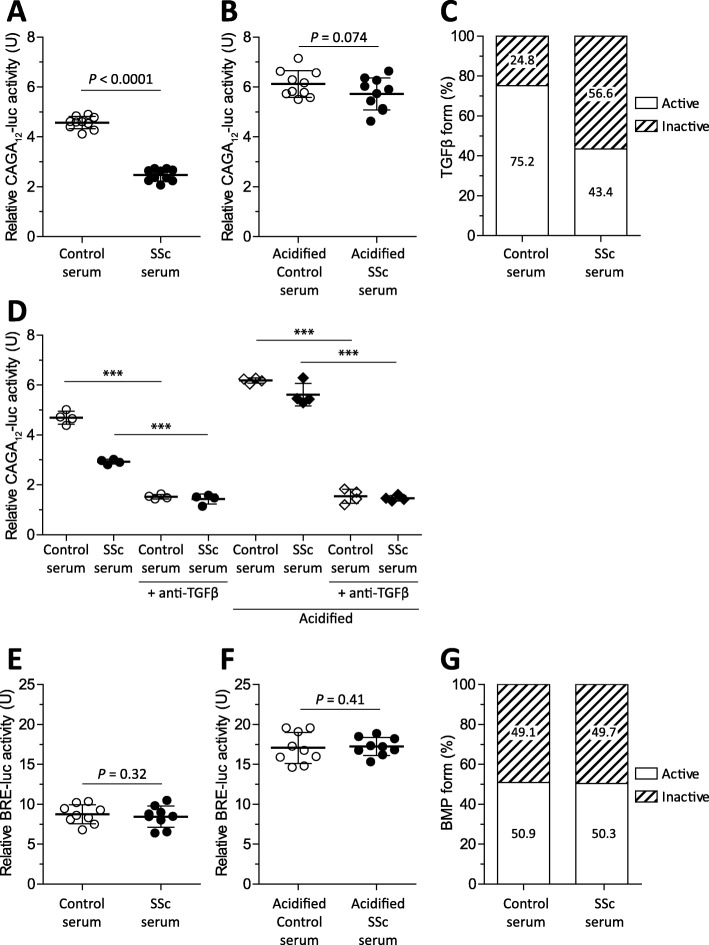
Reduced TGFβ bioactivity of SSc serum on primary human fibroblasts. Primary human fibroblasts expressing a SMAD3-dependent luciferase construct (CAGA_12_-luc) were stimulated with serum (**a**) or acidified serum (**b**) of SSc (*n* = 10) or age- and sex-matched controls (*n* = 10) for 16 h, and luciferase activity was measured. Using these results, TGFβ latency was calculated (**c**). A TGFβ1/2/3 neutralizing antibody was added to both normal and acidified serum to demonstrate the contribution of TGFβ to bioassay readout (**c**). Primary human fibroblasts expressing a SMAD1/5-dependent luciferase construct (BRE-luc) were stimulated with serum (**e**) or acidified serum (**f**) of SSc (*n* = 10) or age- and sex-matched controls (*n* = 10) for 16 h, and luciferase activity was measured. Using these results, BMP latency was calculated (**g**). Statistics of **a**, **b**, **e**, and **f** were calculated using *t* tests and of **d** using ANOVA

To begin with, we determined TGFβ activity in serum of ten SSc patients and age- and sex-matched controls. For this, serum was added to human skin fibroblasts expressing CAGA_12_-luciferase. This CAGA_12_-luc construct produces luciferase in response to c-terminally phosphorylated SMAD3, an intracellular signaling route typically activated by TGFβ. To determine the amount of latent TGFβ, a comparison was made between TGFβ bioactivity in serum versus that in acidified serum. Acidification irreversibly releases TGFβ from its latency-conferring peptides, allowing for determination of total TGFβ levels. By comparing total TGFβ levels to measured TGFβ levels in untreated serum, bioavailability (i.e., latency) of TGFβ can be determined. Remarkably, Fig. 1a shows that TGFβ activity was lower in SSc sera than in control sera; relative CAGA_12_-luciferase activity was significantly ± 50% lower. In contrast, total TGFβ activity, as measured in acidified serum, was not lower, as shown in Fig. 1b. From these data, latency was calculated by division. In control serum, 75.2% of TGFβ was bioactive, whereas in the SSc serum, only 43.4% of TGFβ was bioactive, which thus corresponds to a difference in latency of  24.8 versus 56.6 percent (Fig. 1c). To confirm that the observed CAGA_12_-luciferase activity was TGFβ-dependent, both normal and acidified sera of the four patients was incubated with a TGFβ-neutralizing antibody, and luciferase production was measured. Figure 1d shows again that the SSc serum induced less luciferase activity than the control serum, but not after acidification. However, in both control serum and acidified serum, the use of a TGFβ-neutralizing antibody profoundly inhibited luciferase activity, showing that the induction of CAGA_12_-luciferase activity by serum was indeed largely TGFβ-dependent. We also measured BMP activity in primary skin fibroblasts using BRE-luciferase which produces luciferase in response to BMP-induced SMAD1/5 signaling. BMPs are members of the TGFβ family which share structural homology and a similar mechanism in which activity is controlled by binding of inhibitory peptides, and similarly, these inhibitory proteins can be removed using acidification. In contrast to TGFβ signaling, BMP signaling was not different in the SSc serum; Figs. 1e and f show that neither in normal nor acidified serum a difference in BMP activity was observed between the SSc and control sera, resulting in a similar bioactivity on skin fibroblasts, i.e., 50.9 versus 50.3%. This observation indicates that lowered bioactivity is not a generalized phenomenon in the SSc serum. Together, these experiments show that TGFβ bioavailability is lower in the SSc serum versus in the control serum.

### Lowered integrin expression on SSc monocytes

**Fig. 2:**
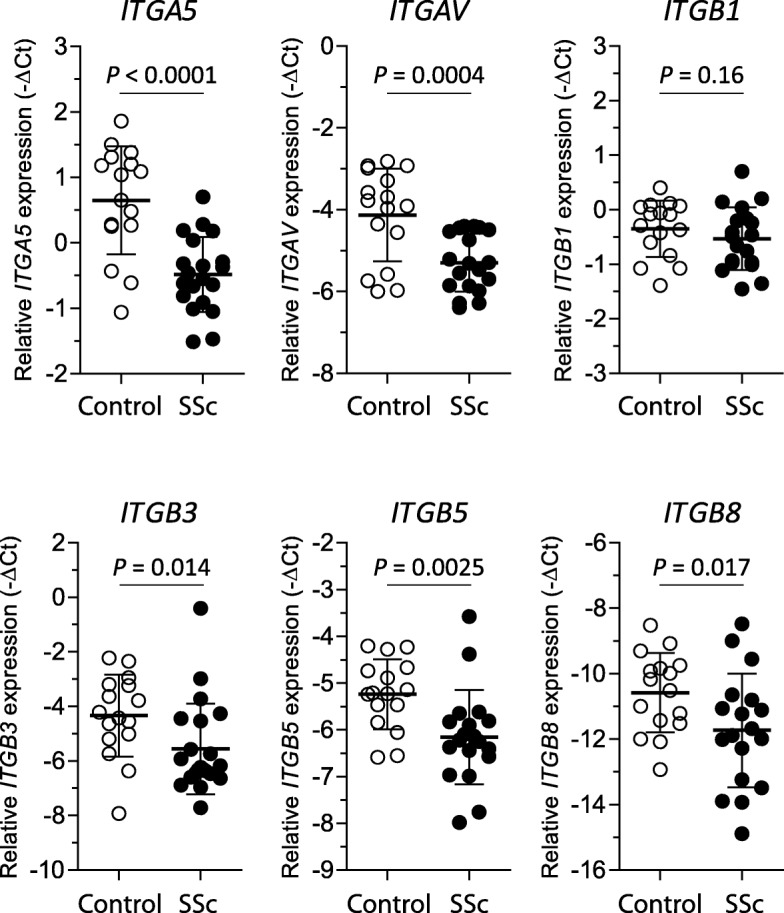
Reduced expression of TGFβ-activating integrins in SSc monocytes. CD14+ monocytes were isolated using positive selection from the PBMC fraction of fresh blood. Immediately after isolation, cells were lysed and RNA extracted. With the use of qPCR, gene expression was measured. Statistics were calculated using Student’s *t* test

Recently, it was shown that monocytes can activate latent TGFβ via an integrin- and MMP14-mediated mechanism and that this activation is key in immune modulation [4]. Therefore, we investigated integrin expression on monocytes of SSc patients (*n* = 20) and healthy donors (*n* = 16). Multiple integrins can be used to activate latent TGFβ, e.g., those composed of *ITGAV*, *ITGA5*, *ITGB1*, *ITGB3*, *ITGB*, *ITGB6*, and *ITGB8* subunits. These integrins can form functional αβ complexes able to recognize arginine-glycine-aspartate (RGD) motifs, which is required for binding of latent TGFβ. Thus, we measured the expression of the aforementioned integrins in freshly isolated CD14+ monocytes (Fig. 2). Strikingly, the gene expression of both alpha subunits was significantly reduced in SSc patients; both *ITGA5* (*P* < 0.0001) and *ITGAV* (*P* < 0.0004) expression was reduced approximately twofold on average compared to healthy controls. Furthermore, *ITGB3* (*P* = 0.01), *ITGB5* (*P* = 0.003), and *ITGB8* (*P* = 0.02) expression was also significantly reduced in SSc monocytes by approximately three-, two-, and threefold respectively. In contrast, *ITGB1* expression was not different, and *ITGB6* expression could not be detected. In conclusion, gene expression of TGFβ-activating integrins is markedly reduced in SSc monocytes.

### Modulation of integrin gene expression by TGFβ1 in SSc monocytes

**Fig. 3:**
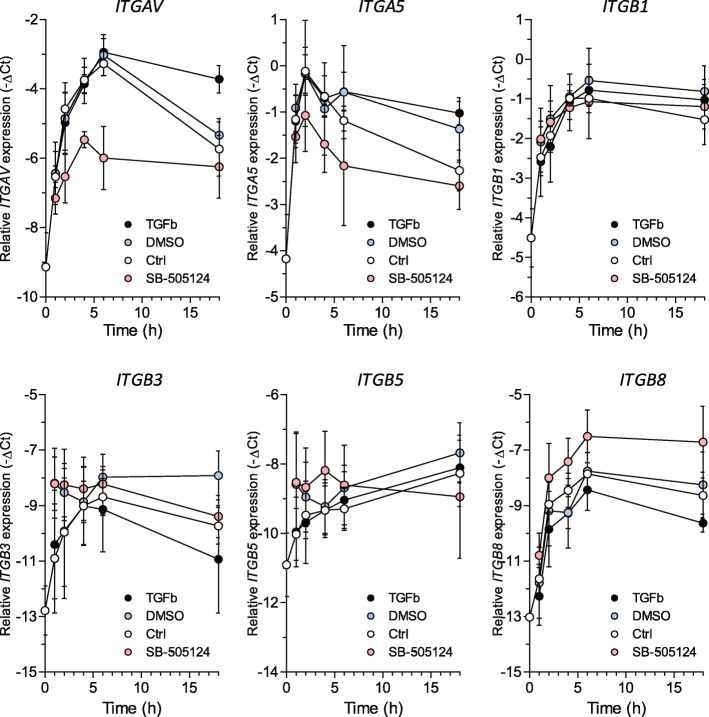
TGFβ regulates the expression of TGFβ-activating integrins in SSc monocytes. CD14+ monocytes were isolated using positive selection from the PBMC fraction of fresh blood and stimulated for 0, 1, 2, 4, 6, and 16 h with 5 ng/ml rhTGFβ1 or the TGFβ receptor inhibitor SB-505124 at a dose of 5 μM or vehicle control (DMSO) while seeded on plastic. At indicated time points, RNA was isolated and gene expression was measured

After observing the reduced integrin expression in SSc monocytes, we investigated if TGFβ levels affect the integrin expression on monocytes. Therefore, we stimulated freshly isolated CD14+ monocytes with either recombinant human TGFβ or a small molecule inhibitor of TGFβ signaling, SB-505124, i.e., an inhibitor of TGFBR1, and studied the effects on integrin expression over time (16 h) (Fig. 3). Directly after isolation, the expression of the integrins *ITGAV*, *ITGA5*, *ITGB1*, *ITGB3*, *ITGB6*, and *ITGB8* strongly increased over time for at least 6 h. This increase likely reflects monocyte activation by plastic adherence. Sixteen hours after stimulation, expression of all measured integrins except ITGB5 was lower than at 6 h. Strikingly, the rapid induction of *ITGAV* and *ITGA5* could be strongly inhibited by the addition of SB-505124, but this TGFBR inhibitor had no effect on the early induction of *ITGB1*, *ITGB3*, and *ITGB5* and even potentiated *ITGB8* induction. The addition of rhTGFβ1 had no effect on early induction of integrin expression, yet after 16 h, the expression of *ITGAV* and *ITGA5* was increased compared to the control. Again, notably, the expression of *ITGB1*, *ITGB3*, and *ITGB5* was not affected, whereas the expression of *ITGB8* was reduced. These data show that TGFβ regulates the expression of integrin α and β subunits differently and even in the opposite fashion for *ITGB8* versus *ITGAV* and *ITGA5*.

## Discussion

In our experiments, we observed that the SSc serum induced less TGFβ/SMAD3-dependent signaling in primary human skin fibroblasts than the healthy control serum, whereas BMP/SMAD1/5-dependent signaling was similar. In circulating CD14+ monocytes, decreased integrin αV and α5 gene expression was observed in the SSc patients, together with reduced integrin β1, β3, β5, and β8 gene expression. Furthermore, we observed that expression of these integrin subunits was regulated by TGFβ, as demonstrated by the use of the TGFBR1 (ALK5) kinase inhibitor SB-505124 and exogenously added hTGFβ1.

Although TGFβ is widely regarded as an important driver of SSc pathophysiology [1], e.g., because it stimulates the excessive production of collagen type I by skin fibroblasts and enhances the contractile properties of these cells [10], its plasma and serum levels have been a controversial topic for many years; studies have reported increased [11–13], equal, and, similarly to our study, decreased levels [14, 15] of TGFβ in serum and plasma of the SSc patients. Possibly, these differences can be attributed to the heterogeneity of disease course in SSc or to the heterogeneity of disease duration in our cohort. However, we measured TGFβ in the serum of patients with long or short (< 2 year) disease duration but observed reduced TGFβ activity in both categories (data not shown). Alternatively, disease activity of patients plays a role, yet it is impossible to compare between studies, as objective measures of disease activity, other than an increase of skin involvement, are lacking to date. Another option is that (enhanced) local activation processes drive pathophysiology, and not circulating TGFβ levels. In this light, our observations on integrin expression can be very relevant, as these proteins are key in TGFβ activation, which is illustrated by the fact that multiple strategies are currently pursued and aimed at inhibiting integrin-mediated TGFβ activation in cancer and fibrosis.

We used a bioassay to determine TGFβ activity in SSc serum in a disease-relevant cell type—the skin fibroblast. In our opinion, the use of bioassays is more valuable than the use of enzyme-linked immunosorbent assays (ELISAs) if the aim is to identify and understand the role of a compound in a disease’s pathophysiology, especially in the case of a growth factor such as TGFβ. Because latency plays such an important role in TGFβ signaling, it is arguably more relevant to determine biological activity versus absolute levels, e.g., if a cell is unable to activate latent TGFβ, high levels of latent TGFβ are biologically irrelevant to that cell. With the use of a bioassay, biological activity can be determined. Of course, with the use of an ELISA also, active levels of TGFβ can be measured [11], but it should be realized that the activity of TGFβ measured in our bioassay not only reflects the presence of active TGFβ in serum, but also how well skin fibroblasts can activate (part of) the latent pool of TGFβ in the course of the experiment (16 h). Furthermore, TGFβ signaling is known to be cellular context dependent, and for example, pro-inflammatory factors present in serum can alter TGFβ signaling. Such context and the ability of cells to handle growth factor latency are integrated in a (well-designed) bioassay and not in an ELISA, leading to biologically more meaningful data from a bioassay.

A possible explanation for the lowered bioactivity of TGFβ we measured in the SSc serum is the increased expression of LTBP4 as was recently identified [13] or the presence of other latency conferring peptides. Alternatively, immune-related compounds present in the serum possibly modulate the abovementioned cellular context of cells in our bioassay and in this way inhibit TGFβ signaling [16]. Irrespective of the mechanism, our data does indicate that the TGFβ in SSc serum is less bioactive on skin fibroblasts. Possibly, this reduction in bioactivity reflects a physiological response of the body to limit excessive TGFβ signaling, which unfortunately fails because the mechanism of increasing TGFβ latency does not work in inflammatory conditions; in these conditions, TGFβ is readily activated, for example, via proteases which degrade latency conferring proteins or reactive oxygen species which disrupt the tertiary structure of latency conferring proteins [17, 18].

Integrins are key in the activation of latent TGFβ. As mentioned, this is illustrated by the phenotype of *itgb6* (integrin β6) and *itgb8* (integrin β8) knockout mice which resembles that of *TGFB1* or *TGFB3* knockout mice [3]. Especially in the immune system [19], integrin-MMP14 activation of TGFβ seems of great importance, in particular via the use of αVβ8 complexes. In both human [20] and murine [21] regulatory T cells, inhibition of αVβ8 integrin complexes leads to an inability to activate TGFβ and loss of immune suppressor function. Also, in human dendritic cells [22], macrophages, and monocytes [4], the importance of αVβ8 complexes in the activation of TGFβ for immune modulation has been demonstrated. Furthermore, in leukocytes, integrins (including RGD-recognizing) are known for their importance in extravasation, and their expression and activation can be induced by pro-inflammatory stimuli, e.g., αVβ8 expression is induced by Toll-like receptor signaling [23] and IL-1 [24]. In view of this, and because systemic sclerosis’ pathophysiology is closely associated with excessive TGFβ signaling [1], we expected an increased expression of TGFβ-activating integrins on monocytes. However, we observed a reduced expression of *ITGAV*, *ITGA5*, *ITGB3*, *ITGB5*, and *ITGB8*. Furthermore, we observed that TGFβ induced *ITGAV* and *ITGA5*, whereas it reduced *ITGB8* expression*.* Induction of *ITGAV* and *ITGA5* by TGFβ has been reported in various cell types before [25], but the reduction of *ITGB8* expression to our knowledge not. Reduced TGFβ activity in the SSc serum and reduced *ITGAV* and *ITGA5* levels in the SSc monocytes are possibly related in view of the ability of active TGFβ to induce (and TGFβ inhibition to lower) the expression of these genes. However, we did not test the direction of this relation, as lower integrin expression on monocytes possibly affects the latency of circulating TGFβ. In contrast, *ITGB8* expression was reduced by TGFβ stimulation and induced by TGFβ inhibition. Because *ITGB8* has been described as a key factor in the TGFβ activation by monocytes [4], these observations possibly indicate a negative feedback mechanism aimed at lowering TGFβ activation. Previously, such a negative feedback mechanism has also been suggested to be present in SSc fibroblasts, via enhanced expression of endoglin, a type III receptor of TGFβ activation which can act as scavenger to lower signaling [26].

## Conclusions

In summary, we described a reduced TGFβ bioactivity of SSc serum on human skin fibroblasts, together with reduced expression of TGFβ-activating integrins in monocytes and regulation of expression of these integrins by TGFβ. These observations support for a role for altered TGFβ bioactivity and changes in the cellular machinery responsible for TGFβ activation in SSc pathophysiology.

## Data Availability

The datasets analyzed in the current study are available from the corresponding author on reasonable request.
